# An enzyme-linked immunosorbent assay (ELISA) for quantification of human collectin 11 (CL-11, CL-K1)

**DOI:** 10.1016/j.jim.2011.10.010

**Published:** 2012-01-31

**Authors:** L. Selman, M.L. Henriksen, J. Brandt, Y. Palarasah, A. Waters, P.L. Beales, U. Holmskov, T.J.D. Jørgensen, C. Nielsen, K. Skjodt, S. Hansen

**Affiliations:** a Department of Cancer and Inflammation Research, Institute of Molecular Medicine, University of Southern Denmark, Winslowparken 21-1, DK-5000 Odense, Denmark; b Molecular Medicine Unit, Institute of Child Health, University College London, 30 Guilford Street, London WC1N 1EH, UK; c Institute of Molecular Medicine, University of Southern Denmark, Winslowparken 25-3, DK-5000 Odense, Odense, Denmark; d Department of Biochemistry and Molecular Biology, University of Southern Denmark, Campusvej 55, DK-5230 Odense M, Denmark; e Department of Clinical Immunology, Odense University Hospital, Sdr. Boulevard 29, DK-5000 Odense C, Denmark

**Keywords:** CL-11, collectin 11 (also known as CL-K1, collectin kidney 1), MASP-1/3, Mannose-binding lectin associated serine proteases 1/3, MBL, mannose-binding lectin, SLE, systemic lupus erythematosus, SP-D, surfactant protein D, ELISA, enzyme-linked immunosorbent assay, CRD, carbohydrate recognition domain, SP-A, surfactant protein A, SP-D, surfactant protein D, MAb, monoclonal antibody, BSA, bovine serum albumin, OD, optical density, QC, quality control, CV, coefficient of variation, Collectin 11, Collectin kidney 1, Complement, Innate immunity, Enzyme-linked immunosorbent assay, 3MC syndrome

## Abstract

Collectin 11 (CL-11), also referred to as collectin kidney 1 (CL-K1), is a pattern recognition molecule that belongs to the collectin group of proteins involved in innate immunity. It interacts with glycoconjugates on pathogen surfaces and has been found in complex with mannose-binding lectin-associated serine protease 1 (MASP-1) and/or MASP-3 in circulation. Mutation in the CL-11 gene was recently associated with the developmental syndrome 3MC. In the present study, we established and thoroughly validated a sandwich enzyme-linked immunosorbent assay (ELISA) based on two different monoclonal antibodies. The assay is highly sensitive, specific and shows excellent quantitative characteristics such as reproducibility, dilution linearity and recovery (97.7–104%). The working range is 0.15–34 ng/ml. The CL-11 concentration in two CL-11-deficient individuals affected by the 3MC syndrome was determined to be below 2.1 ng/ml. We measured the mean serum CL-11 concentration to 284 ng/ml in 100 Danish blood donors, with a 95% confidence interval of 269–299 ng/ml. There was no significant difference in the CL-11 concentration measured in matched serum and plasma samples. Storage of samples and repeated freezing and thawing to a certain extent did not influence the ELISA. This ELISA offers a convenient and reliable method for studying CL-11 levels in relation to a variety of human diseases and syndromes.

## Highlights

► The ELISA is based on monoclonal antibodies, highly sensitive and specific, and is the first of its kind. ► Validation shows excellent reproducibility, dilution linearity and recovery. ► We find that mean serum CL-11 concentration in 100 blood donors is 284 ng/ml (95%CI = 269–299 ng/ml). ► The ELISA allows for direct comparison of plasma and serum samples. ► We measure the CL-11 concentration in persons with 3MC syndrome to be less than 2.1 ng/ml.

## 1 Introduction

Collectin 11 (CL-11), also known as collectin kidney 1 (CL-K1), belongs to the collectin group of the innate immune molecules structurally characterized by containing a carbohydrate recognition domain and a collagen-like region (Keshi et al., 2006). CL-11 is ubiquitously expressed, but highest levels are found in the adrenal glands, the kidneys, and the liver, and it is also present in circulation (Hansen et al., 2010). It is highly conserved among species ranging from zebrafish to humans. CL-11 has been shown to bind to intact bacteria, fungi and influenza A virus, and also to decrease influenza A infectivity. CL-11 was found to be associated with mannose-binding lectin-associated serine protease 1 (MASP-1) and/or MASP-3 in plasma (Hansen et al., 2010). These findings indicate a role for CL-11 in the defense against pathogens and in the activation of the complement system. Recently, CL-11 and MASP-3 were shown to be involved in fundamental developmental processes. Individuals with mutations in genes encoding either of the two proteins display a wide spectrum of developmental disorders known as the 3MC syndrome (Rooryck et al., 2011).

Variations in serum levels of collectins have been implicated in various immunity disorders. Functional mannose-binding lectin (MBL) deficiency, caused by single-nucleotide polymorphisms in the coding region of the *MBL2* gene, has been associated with increased susceptibility to infections in young children and immunocompromised individuals (Sumiya et al., 1991; Garred et al., 1997; Summerfield et al., 1997; Neth et al., 2001; Peterslund et al., 2001). MBL deficiency or low MBL serum levels are also associated with the occurrence of autoimmune disorders, such as systemic lupus erythematosus (SLE) (Lee et al., 2005). Circulatory levels of the otherwise lung-associated collectin surfactant protein D (SP-D) are increased upon lung injuries (Leth-Larsen et al., 2003). Low serum levels of SP-D, caused by the variant allele Thr11, may increase susceptibility to tuberculosis (Floros et al., 2000). Low serum levels of SP-D have also been implicated in pathogenesis of SLE (Hoegh et al., 2009).

In order to identify the biological functions of CL-11, it is necessary to be able to measure CL-11 levels in serum and other fluids. The objectives of the present work were to develop and validate an enzyme-linked immunosorbent assay (ELISA) for measuring human CL-11 in various samples, and to determine CL-11 levels in normal serum and plasma.

## 2 Materials and methods

### 2.1 Reagents and buffers

Unless otherwise stated, reagents were obtained from Sigma-Aldrich (Vallensbaek, Denmark). The following buffers were used: TBS: (10 mM Tris and 145 mM NaCl, pH 7.4), coating buffer (15 mM Na_2_CO_3_, 35 mM NaHCO_3_, pH 9.6), washing buffer for ELISA (TBS, 5 mM EDTA, 0.05% Emulfogen, pH 7.4), substrate buffer (35 mM citric acid, 67 mM Na_2_HPO_4_, 0.012% H_2_O_2_, pH 5.0), washing buffer for Western blotting (TBS, 5 mM EDTA, 0.1% Emulfogen, 5% non-fat dried milk, 0.1% w/v BSA, pH 7.4).

### 2.2 Expression and purification of recombinant CL-11

The expression and purification of recombinant CL-11 were performed as previously described (Hansen et al., 2010). Briefly, full-length untagged human CL-11 was expressed in DG44 CHO cells using the bicistronic pOptiVEC TOPO vector (Invitrogen, Taastrup, Denmark). Recombinant CL-11 was purified from the culture supernatant using mannose-Sepharose affinity purification. The concentration of CL-11 was measured by quantitative amino acid analysis of 7 different fractions of purified CL-1 from three different rounds of purification. The derived average conversion factor of the 7 analyses was used throughout the study.

### 2.3 Anti-CL-11 MAbs

Monoclonal antibodies (MAbs) were essentially produced by the principles described by Kohler and Milstein (Kohler and Milstein, 1975) in outbred NMRI mice with modifications previously described (Hansen et al., 2008). Briefly, purified recombinant CL-11 was used as the antigen. Positive clones were identified by ELISA using microtiter plates coated with CL-11. Cells from the positive wells were cloned at least four times by limiting dilution. For MAb production and subsequent purification, hybridomas were grown and allowed to express the MAbs in Hybridoma-SFM with 0.75% ultra-low IgG fetal bovine serum (both from Invitrogen). MAbs were purified by means of affinity chromatography using a HiTrap Protein G HP column (GE Healthcare, Piscataway, NJ USA) under previously described conditions (Akerstrom and Bjorck, 1986). MAbs were biotinylated using biotin *N*-hydroxysuccinimide ester according to the manufacturer's recommendations. Briefly, purified antibodies were dialyzed against 0.1 M carbonate buffer, pH 8.5, and biotin *N*-hydroxysuccinimide ester was added corresponding to 1/6 (w/w) of the total protein amount. The mixture was incubated with gentle mixing for 4 h at room temperature. Unreacted biotinylation reagent was removed by dialysis against TBS, pH 7.4.

### 2.4 SDS-PAGE and Western blotting

Proteins were separated by SDS-PAGE in 4–12% Novex Bis-Tris gels (Invitrogen) and blotted onto polyvinylidene difluoride membranes (PVDF HyBond, Amersham Biosciences, Little Chalfont, UK). The membranes were washed and blocked for 1 h in washing buffer, followed by overnight incubation at 4 °C with biotinylated primary MAbs (0.52 μg/ml of 11–2, 1.0 μg/ml of 14–29 or 1.0 μg/ml of isotype-matched (IgG1κ) control anti-mouse SP-D K403) diluted in washing buffer. After repeated washing, the membranes were incubated for 1 h at room temperature in horseradish peroxidase (HRP)-conjugated Streptavidin (Invitrogen) diluted to 1/10,000 in washing buffer. The membranes were washed and developed with aminoethyl carbazol.

### 2.5 ELISA for CL-11 and assay optimization

Polystyrene microwell plates (Maxisorp, Nunc, Roskilde, Denmark) were coated with 5 μg MAb 11–2 per ml coating buffer (100 μl/well). After overnight incubation at 4 °C, the coated wells were washed three times and left to block with washing buffer for 30 min at room temperature. The calibrator, controls and samples were diluted in washing buffer containing bovine serum (0.1% v/v; AH diagnostics, Aarhus, Denmark) and heat-aggregated human IgG (50 μg/ml; Innovative Research, Novi, MI, USA) and incubated overnight at 4 °C. Subsequently, the wells were washed three times and biotinylated MAb 14–29 diluted to 0.5 μg/ml in washing buffer containing BSA (1 mg/ml) was added to the wells and incubated for 1 h at room temperature. After three washes, HRP-conjugated Streptavidin diluted to 1/12,000 in washing buffer containing BSA (1 mg/ml) was added to the wells and incubated for 30 min at room temperature. The wells were washed three times and 0.4 mg of *o*-phenylene-diamine (Kem-En-Tec, Taastrup, Denmark) was added per ml substrate buffer. After 15 min the color development was stopped with 1 M H_2_SO_4_. Optical density (OD) was measured at 490–650 nm using Vmax Kinetic Microplate Reader and the data were processed using SoftMax Pro software (Molecular Devices, Wokingham, United Kingdom). The samples were diluted to 1/40 and the calibrator, quality controls (QCs) and samples were run in triplicates unless otherwise stated. Optimal dilutions of MAb 11–2 and biotinylated MAb 14–29 were determined by standard checkerboard titrations of the dilution of one MAb against the dilution of plasma. The optimal concentration of HRP-conjugated streptavidin was determined in the same way.

### 2.6 Assay validation

#### 2.6.1 Calibrator and quality controls

The calibrator consisted of the culture supernatant from DG44 CHO cells expressing recombinant CL-11. A two-fold serial dilution of the culture supernatant was used to generate an eight-point calibrator curve with a range from 0.26 to 34.8 ng/ml. A five-parameter fit model was applied to the calibrating samples and used to estimate the concentration of unknown samples. The calibrator was stored as single-use aliquots at − 80 °C. The QCs consisted of a pool of serum or plasma from five healthy volunteers diluted 1/11, 1/80 and 1/500 in dilution buffer to represent high, medium and low concentrations of CL-11, respectively. The QCs were stored as single ready-to-use aliquots at − 80 °C.

#### 2.6.2 Parallelism

To study parallelism, the calibrator serial dilution curve was compared to the serial dilution curves of two batches of purified recombinant CL-11 and serial dilutions curves of plasma and serum from two blood donors (analyzed in duplicates). OD data were evaluated using regression analysis on logistically transformed values, an algorithm that comprised several steps. Due to the maximum limit of the OD determination, a number of consecutive measurements of OD = 4.0 was observed in each dilution series. Only the last value of OD = 4.0 was maintained in each dilution series, while the prior maximum determinations were omitted. Subsequently, all OD values were divided by 4.1 to transform the OD data to values above 0, but below 1, as required for the subsequent logistic transformation, y′ = ln[y/(1 − y)]. A background level of OD = 0.05 was observed, and values below the corresponding logistically transformed values were omitted from further analysis. A linear regression was fitted to the remaining data points and multiple comparisons among slopes using Tukey's HSD test were used to compare the parallelism of the different serial dilutions. The statistical analyses were performed using the Analyse-it software (Analyse-it Software, Ltd, Leeds, UK).

#### 2.6.3 Working range and detection limit

Ten two-fold serial dilutions of serum and plasma samples from five blood donors were analyzed in triplicates. Coefficients of variation (CV) were calculated for the triplicate measurements of each dilution. A “measured/mean” ratio was expressed for each sample using the triplicate measurements and calculating the mean of the triplicates. To study linearity, the CL-11 concentration calculated for each dilution and multiplied by the dilution factor was compared to a mean of the CL-11 concentration that was back-calculated from four dilutions of each sample (1/16–1/128 for serum samples and 1/20–1/160 for plasma samples). The working range was determined as the CL-11 concentrations for which CV was < 10% and the measured/mean ratios deviated < 20%. The lower detection limit was calculated as the background signal ± 2 standard deviations (SD).

#### 2.6.4 Recovery

Serum and plasma samples from three healthy volunteers were diluted to contain different levels of endogenous CL-11 and spiked with DG44 CHO cell culture supernatant containing recombinant CL-11. Recovery was calculated as the ratio of measured CL-11 over the expected total CL-11 concentration.

#### 2.6.5 Intra- and interassay variation

Intraassay variation was calculated by running the QCs in 22 replicates on a single plate. The interassay variation was determined by running the QCs in triplicates on ten plates on five separate occasions. Intra- and interassay CVs < 10% were found acceptable.

### 2.7 Storage temperature and effect of repeated freezing and thawing

Serum and plasma samples (250 μl aliquots) from five different healthy persons were stored at room temperature, 4 °C and − 20 °C for 1 week. CL-11 levels were measured after 24 h and after 1 week of storage. Furthermore, CL-11 was measured in samples stored at − 20 °C and − 80 °C for one month. The fresh sample aliquots were also subjected to eight freeze-thaw cycles (− 20 °C and room temperature, respectively) and CL-11 levels were measured after 1, 2, 3, 4 and 8 freeze-thaw cycles.

### 2.8 Serum and plasma samples

Matched serum and EDTA-plasma samples collected from 100 Danish blood donors and serum samples from two individuals affected by 3MC syndrome, who carry a homozygous mutation in *COLEC11* (p.Gly204Ser), were tested in ELISA in triplicates at a dilution of 1/40 and 1/14, respectively. The normality of the data was evaluated using the Shapiro–Wilk test. The Altman–Bland method was used to assess differences in CL-11 concentrations between the matched serum and plasma samples. EDTA-plasma from two healthy individuals was depleted for CL-11 by passage through an anti-CL-11 MAbs column (4 different anti-CL-11 MAbs conjugated to Sepharose) and tested in ELISA in triplicates at a dilution of 1/10 or 1/20.

## 3 Results

### 3.1 Characterization of monoclonal antibodies and optimization of ELISA

**Fig. 1:**
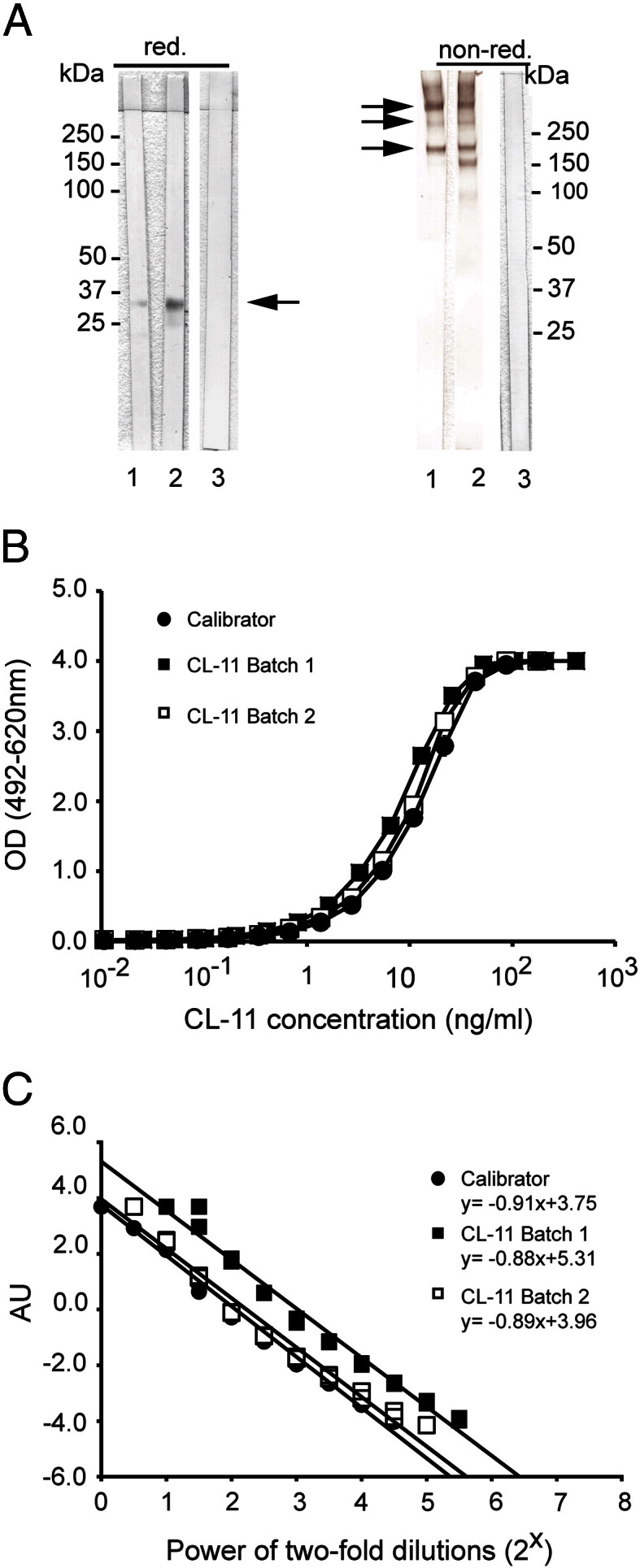
Characterization of MAbs 11–2 and 14–29 and calibration of the ELISA. (A) Western blotting of serum preincubated into ELISA wells coated with MAb 11–2 (10 μg/ml) and eluted with SDS-PAGE loading buffer under reducing (red.) and non-reducing conditions (non-red.). Blots were developed with biotinylated MAbs 14–29 (1), 11–2 (2) or the control MAb (3). (B) A calibrator curve was made of serial dilutions of culture supernatant from cells expressing recombinant CL-11. It was compared with serial dilutions of two batches of purified recombinant CL-11. The CL-11 concentration of purified CL-11 was adjusted to best fit with OD values of the calibrator. The samples were tested in duplicates. (C) To analyze parallelism, a linear regression was fitted to logistically transformed OD values (R^2^ > 0.97 for all curves) and used for comparisons of the slopes (see Materials and methods). The logistically transformed OD values are presented in arbitrary units (AU).

The specificity of MAbs 11–2 and 14–29 was analyzed by Western blotting. To mimic the ELISA setup, bound serum antigens were eluted from microtiter wells coated with MAb 11–2 and analyzed by Western blotting using biotinylated MAbs 14–29 and 11–2 (Fig. 1A). By this approach, a protein band of 34 kDa, corresponding to full-length CL-11, was detected in reduced eluates. The biotinylated MAb 14–29 reacted only weakly with reduced CL-11. Under nonreduced conditions immunoreactivity bands at 200 and 300 kDa were detected, corresponding to dimers and trimers of subunits of CL-11, as well as several oligomers larger than 300 kDa. In addition, a faint band of approximately 28 kDa (not detected with MAb 14–29) and a band of approximately 160 kDa were detected in the reduced and nonreduced eluates, respectively. These bands also developed with other anti-CL-11 MAbs (data not shown) and therefore we speculate that they also represent CL-11 (see discussion). From a panel of 50 mouse anti-human CL-11 MAbs recognizing at least seven different epitopes of CL-11, MAb 11–2 and biotinylated 14–29 were chosen for capture and detection, respectively. The MAbs bind to different epitopes on CL-11 and MAb 11–2 requires the presence of EDTA for optimal binding activities (data not shown). Judged by the highest signal-to-noise ratio and maximum read-out signal, this combination of MAbs resulted in a sandwich ELISA with highest sensitivity. The ELISA was further optimized in terms of conditions and concentrations of MAb 11–2, biotinylated MAb 14–29, HRP-Streptavdin and additives (BSA, heat-aggregated IgG and bovine serum; data not shown).

### 3.2 Validation of ELISA

**Fig. 2:**
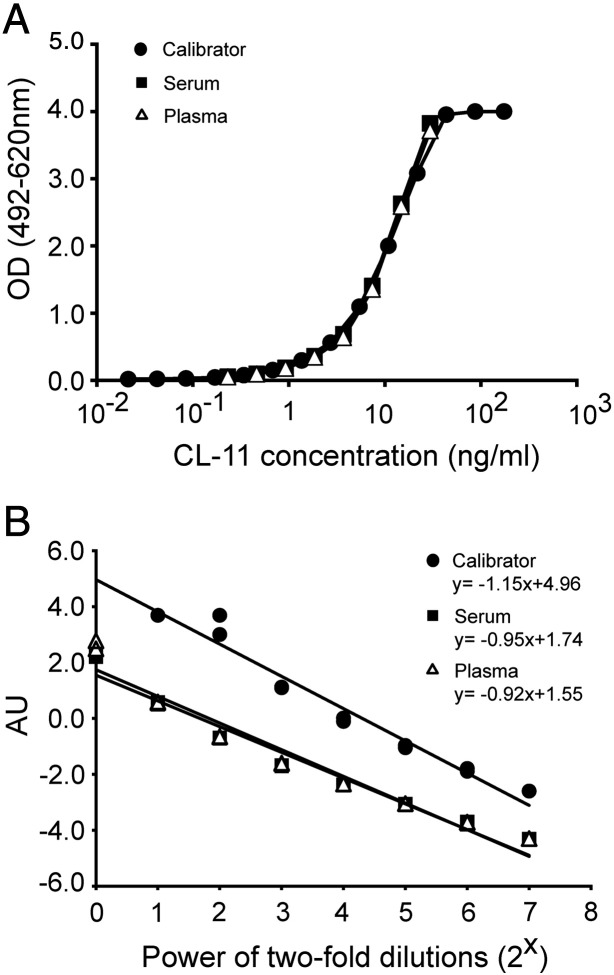
Validation of ELISA: parallelism between the calibrator and the samples. (A) Comparison of the calibrator curve with serially diluted serum and plasma samples. The CL-11 concentration in serum and plasma was adjusted to best fit with OD values of the calibrator. The samples were tested in duplicates and the analysis is representative of comparison with samples from two donors. (B) Regression analysis on logistically transformed OD values (R^2^ > 0.95 for all curves) was performed and used for comparisons of the slopes (see Materials and methods). The logistically transformed OD values are presented in arbitrary units (AU).

**Fig. 3:**
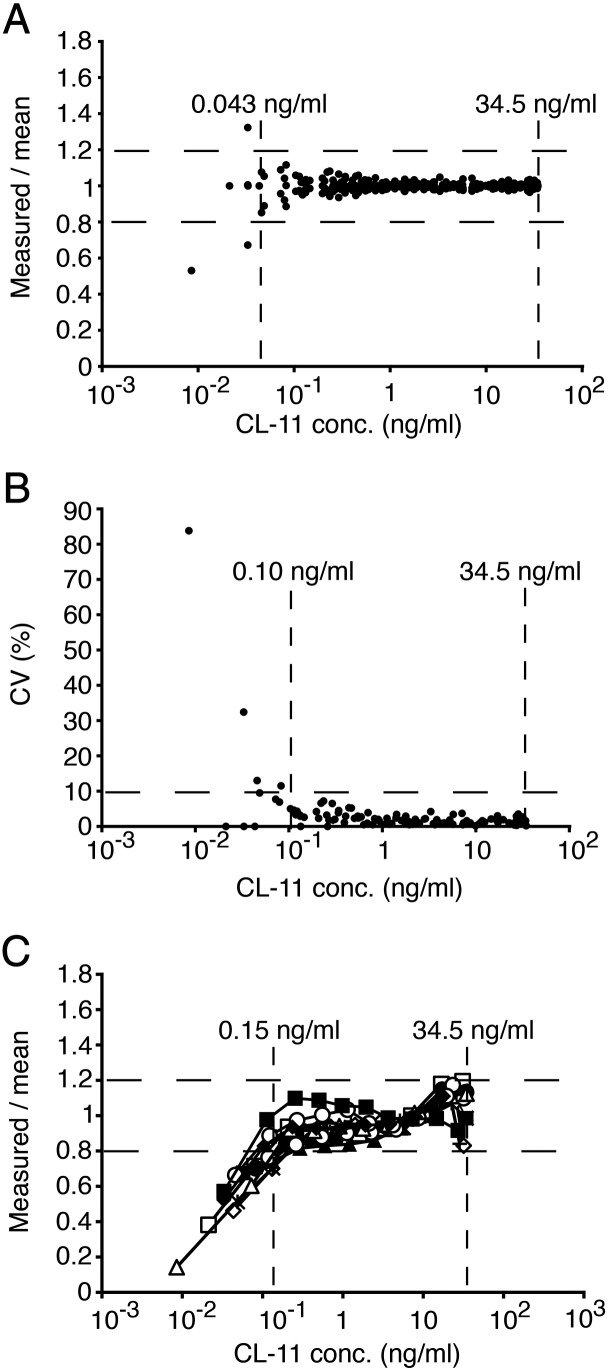
Validation of ELISA: precision and linearity analysis. Serial dilutions of serum and plasma samples from five blood donors were analyzed in triplicates. (A) The measured/mean concentration ratio as a function of CL-11 concentration. The range of 0.04–34.5 ng/ml corresponds to an acceptable deviation of < 20%. (B) The CV(%) as a function of CL-11 concentration. The range of 0.10–34.5 ng/ml corresponds to an acceptable CV of < 10%. (C) The linearity of CL-11 concentrations. The range of 0.15–134.5 ng/ml corresponds to an acceptable deviation of < 20%.

Parallelism was observed between the serial dilution curves of the calibrator and two batches of purified recombinant CL-11 (Fig. 1B). Following logistic transformation, the data sets fitted a linear regression with R^2^ > 0.97 for all curves with the slopes between − 0.88 and − 0.91 (Fig. 1C). A Tukey's HSD test revealed that slopes of the serial dilution curves did not differ significantly from each other (p < 0.05). A similar analysis of dilution curves of the calibrator, the serum and the plasma showed also parallelism with slopes between − 0.92 and − 1.15 that did not differ significantly (p < 0.05; Fig. 2). We also observed satisfactory parallelism between dilution curves of the calibrator and serum from two individuals with rheumatoid arthritis. This confirmed that the ELISA was free of interference from rheumatoid factors (data not shown). The working range was based on combinatory evaluation of the coefficient of variation (CV), the measured/mean ratio and the linearity of the dilution curves for serum and plasma from 5 blood donors (Fig. 3). CV was acceptable (< 10%) in the range 0.10 ng/ml–17.1 ng/ml and the measured/mean ratio was acceptable (< 20% deviation from mean) in the range 0.04 ng/ml–34.5 ng/ml. The linearity of diluted samples was found acceptable (< 20% deviation from mean) in the range 0.15 ng/ml–34.5 ng/ml. Based on these findings, the working range of the ELISA was determined to be 0.15–34.5 ng/ml. The lower detection limit was found to be 0.01 ng/ml. The intraassay CVs were determined for both serum- and plasma-derived QCs and varied between 1.7% and 4.8%. The interassay CVs for these samples varied between 5.0% and 8.4%. The validation data are summarized in Table 1. The recovery was assessed by the ability to recover known amounts of recombinant CL-11. The assay recovered 97.7–104% of the expected amounts at working concentrations from 0.26 to 31.3 ng/ml (Table 2).

### 3.3 Levels of CL-11 in serum and plasma

**Fig. 4:**
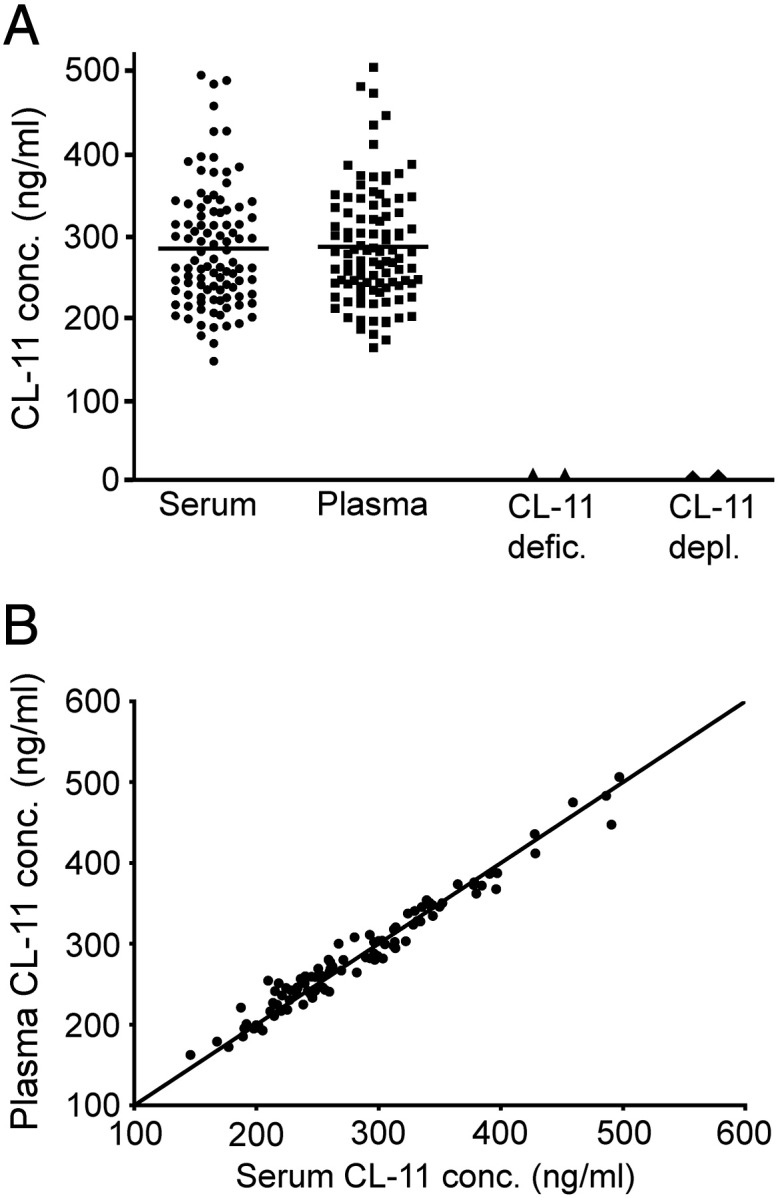
Assessment of CL-11 levels. (A) The CL-11 concentration was measured in matched serum and plasma samples from 100 Danish blood donors. The mean serum CL-11 level is 284 ng/ml with a 95% confidence interval of 269–299 ng/ml. The mean CL-11 levels in serum and plasma are indicated with a solid line. CL-11 concentrations in serum from 3MC syndrome affected individuals (CL-11 defic.) and in CL-11-depleted plasma (CL-11 depl.) were below the lower working limit of the assay (corresponding to < 2.1 ng/ml). (B) Correlation between serum and plasma CL-11 levels in the donors. The solid line indicates the line of identity (x = y).

The CL-11 concentration was determined in matched serum and plasma samples from 100 Danish blood donors (Fig. 4A). The mean serum concentration was estimated to 284 ng/ml with a 95% confidence interval of 269–299 ng/ml and a range of 146–497 ng/ml. There was no significant difference in the CL-11 levels between matched serum and plasma samples (p = 0.15; Fig. 4B). Upon log transformation of data, CL-11 levels in serum and plasma followed a normal distribution (p = 0.62 for serum and p = 0.81 for plasma; data not shown). Using the normally distributed data, we found no significant difference in CL-11 levels between males and females (data not shown). The CL-11 concentration in serum from two CL-11-deficient individuals was below the lower working limit of the assay. Thus, the CL-11 concentration in these sera was lower than 2.1 ng/ml when the dilution of the samples was taken into account (Fig. 4A). Similar observations were made for normal plasma depleted by affinity chromatography on anti-CL-11 MAb columns.

### 3.4 Effect of storage and freezing and thawing

The stability of CL-11 in serum and plasma upon storage at different temperatures was examined using matched serum and plasma samples from five blood donors. Storage of the samples for up to 1 week at room temperature, 4 °C or − 20 °C did not affect the concentration of CL-11 significantly. The CL-11 concentration was not influenced by up to eight freeze/thaw cycles (data not shown).

## 4 Discussion

A quantitative sandwich CL-11 ELISA was developed using two different MAbs and using culture supernatant containing recombinant CL-11 as the calibrator. The current ELISA was thoroughly validated and appears very reliable and robust. Our validation is summarized in Tables 1 and 2, and based on our observations, the ELISA is unaffected by the presence of rheumatoid factors or differences in storage conditions of samples. The ELISA allows also measured serum and plasma concentrations to be directly compared with each other. We observed excellent parallelism between our calibrator made of recombinant CL-11 and plasma and serum samples in all our experiments. We have previously estimated the mean CL-11 concentration to approximately 2 μg/ml in the plasma of 10 healthy individuals based on an absolute correlation with OD_280_ measurements of purified recombinant CL-11 (Hansen et al., 2010). Our quantitative amino acid analysis in present work showed that this was highly overestimated, most likely due to the presence of glycosides that influences the absorbance measurements (not shown). Due to this discrepancy, seven different quantitative amino acid analyses were performed, and the average of these was used for final correlation to absolute concentrations. Antibody specificities were tested by means of Western blotting and we observed only immunoreactivity with bands that we expect to be CL-11. We speculate that the immunoreactive bands at approximately 28 and 160 kDa, in the reduced and nonreduced eluate, respectively, represent the reported CL-11 isoforms that lack parts of the collagen-like region (Keshi et al., 2006) or degraded CL-11 (Fig. 1A). Nonreduced, CL-11 appears to be made of dimers (200 kDa), trimers (300 kDa) and several multimers of subunits larger than the trimers (> 300 kDa). In comparison, previous analysis of recombinant CL-11 showed only the presence of dimers of subunits (Hansen et al., 2010), but judged from the careful studies of parallelism the discrepancy appears not to influence the ELISA. The specificity of the assay was further demonstrated by showing that the CL-11 concentration in serum from two individuals affected by 3MC syndrome was below the lower working limit of the assay. Thus, the levels in these individuals are less than 2.1 ng/ml, comprising less than 0.8% of the mean CL-11 level (284 ng/ml) found in the 100 Danish blood donors. Hence, the ELISA is not influenced by cross reactivity and is also suitable for identifying individuals with CL-11 polymorphisms and altered serum and plasma concentrations. The two individuals affected by 3MC syndrome are homozygous for the same CL-11 polymorphism, characterized by a single nucleotide substitution, c.610 G > A, which results in the amino acid substitution p.Gly204Ser in the carbohydrate recognition domain (Rooryck et al., 2011). The observed deficiency suggests that the substitution leads to retention or instability of CL-11. During the submission of this paper, a study by Wakamiya and colleagues reported an average CL-11 plasma concentration of 340 ± 130 ng/ml in healthy Japanese donors using a combination of polyclonal- and monoclonal-based ELISA (Yoshizaki et al., in press). These findings fall well in line with the mean CL-11 concentration of 284 ng/ml measured in the Danish blood donors. Mutations in the CL-11 and MASP-3 genes were recently linked with the 3MC syndrome, and CL-11 and MASP-3 were shown to play a role in embryonic developmental processes. The functional role of CL-11 in innate immunity requires further characterizations but the interaction with both MASP-1 and MASP-3 implies that it plays a role in the activation of the complement system (Hansen et al., 2010). Recently, MASP-1 was shown to influence activation of factor D and activity of the alternative pathway in mice (Takahashi et al., 2010). In summary, we have established a sandwich ELISA for measuring CL-11 concentrations in human serum and plasma. The ELISA enables evaluation of CL-11 levels in relation to diseases and syndromes. It is our hope that the ELISA and derived reagents will allow for assessment of the functional role of CL-11.

## Figures and Tables

**Table 1:** Assay validation.

	Serum QC			Plasma QC		
	32.5 ng/ml	3.74 ng/ml	0.54 ng/ml	28.3 ng/ml	3.34 ng/ml	0.60 ng/ml
Intraassay var. CV (%)^a^	3.2	1.7	2.5	4.8	3.6	3.8
Intraassay var. CV (%)^b^	7.7	6.6	7.0	5.8	5.0	8.4
Working range (ng/ml)	0.15–34.5					
Detection limit (ng/ml)	0.01					

**Table 2:** Assay validation recovery.

Sample source	Serum	Plasma	Serum	Plasma	Serum
Sample with CL-11 (ng/ml)	31.3 (± 0.30)	26.3 (± 0.59)	5.86 (± 0.12)	1.23 (± 0.01)	0.26 (± 0.01)
Spiking with CL-11 (ng/ml)	3.58 (± 0.02)^a^	3.62 (± 0.03)^a^	3.58 (± 0.02)^a^	3.62 (± 0.03)^a^	3.62 (± 0.03)^a^
Expected (ng/ml)	34.9	29.9	9.44	4.85	3.88
Measured (ng/ml)	35.0 (± 1.03)	30.1 (± 0.45)	9.22 (± 0.05)	4.77 (± 0.05)	4.05 (± 0.03)
Recovery (%)	100 (± 3.0)	101 (± 1.5)	97.7 (± 0.5)	98.4 (± 1.2)	104 (± 0.9)

a n = 6.
